# Ovine reference materials and assays for prion genetic testing

**DOI:** 10.1186/1746-6148-6-23

**Published:** 2010-04-30

**Authors:** Michael P Heaton, Kreg A Leymaster, Theodore S Kalbfleisch, Brad A Freking, Timothy PL Smith, Michael L Clawson, William W Laegreid

**Affiliations:** 1 USDA, ARS, United States Meat Animal Research Center (USMARC) State Spur 18D, P.O. Box 166, Clay Center, NE 68933, USA; 2 University of Louisville, Center for Genetics and Molecular Medicine, 580 South Preston Street, Louisville, KY 40202, USA; 3 University of Illinois, Department of Veterinary Pathobiology, 2001 S. Lincoln Avenue, Urbana, IL 61802, USA

## Abstract

**Background:**

Genetic predisposition to scrapie in sheep is associated with several variations in the peptide sequence of the prion protein gene (*PRNP*). DNA-based tests for scoring *PRNP *codons are essential tools for eradicating scrapie and for evaluating rare alleles for increased resistance to disease. In addition to those associated with scrapie, there are dozens more *PRNP *polymorphisms that may occur in various flocks. If not accounted for, these sites may cause base-pair mismatching with oligonucleotides used in DNA testing. Thus, the fidelity of scrapie genetic testing is enhanced by knowing the position and frequency of *PRNP *polymorphisms in targeted flocks.

**Results:**

An adaptive DNA sequencing strategy was developed to determine the 771 bp *PRNP *coding sequence for any sheep and thereby produce a consensus sequence for targeted flocks. The strategy initially accounted for 43 known polymorphisms and facilitates the detection of unknown polymorphisms through an overlapping amplicon design. The strategy was applied to 953 sheep DNAs from multiple breeds in U.S. populations. The samples included two sets of reference sheep: one set for standardizing *PRNP *genetic testing and another set for discovering polymorphisms, estimating allele frequencies, and determining haplotype phase. DNA sequencing revealed 16 previously unreported polymorphisms, including a L237P variant on the F_141 _haplotype. Two mass spectrometry multiplex assays were developed to score five codons of interest in U.S. sheep: 112, 136, 141, 154, and 171. Reference tissues, DNA, trace files, and genotypes from this project are publicly available for use without restriction.

**Conclusion:**

Identifying ovine *PRNP *polymorphisms in targeted flocks is critical for designing efficient scrapie genetic testing systems. Together with reference DNA panels, this information facilitates training, certification, and development of new tests and knowledge that may expedite the eradication of sheep scrapie.

## Background

Transmissible spongiform encephalopathies (TSEs), or prion diseases, are fatal neurological disorders of humans and other mammals that are characterized by accumulation of an abnormal, protease-resistant isoform of the prion protein in the brain. Naturally occurring prion diseases may have acquired, inherited, or sporadic origins (i.e., no known environmental or genetic cause). TSE outbreaks have arisen in several species and include Creutzfeldt-Jakob disease (CJD) and kuru in humans, bovine spongiform encephalopathy (BSE) in cattle, scrapie in sheep and goats, chronic wasting disease in deer and elk, feline spongiform encephalopathy in cats, and transmissible mink encephalopathy in farmed mink (for review see [1]). Cattle with BSE have been implicated as the cause of one human TSE, variant CJD, through the consumption of beef from affected animals [2-5]. Orally-acquired BSE has also been implicated as a cause of BSE in captive wild animals including: big cats, nonhuman primates, spiral-horned antelope, oryx, and bison [6,7]. Thus, cross-species transmission of TSEs may extend across subfamilies and superorders. Although there is no evidence of sheep scrapie transmission to humans in more than 250 years of exposure [8], uncertainties associated with species barriers have prompted many countries to develop policies aimed at eliminating all TSE-affected animals from their food chains, including scrapie in sheep.

In sheep, distinct prion protein (PrP) isoforms are associated with differences in scrapie susceptibility or disease progression. Increased resistance to classical scrapie is associated with a prion protein gene (*PRNP*) haplotype allele encoding alanine (A), arginine (R), and R at codon positions 136, 154, and 171, respectively (i.e., ARR). Conversely, a haplotype encoding valine (V), R, and glutamine (Q) at those positions (i.e., VRQ) is associated with increased susceptibility or attack rate [9-11]. Haplotype alleles encoding three other forms of PrP (ARQ, AHQ, and ARH, where H is histidine) have intermediate or unknown associations with classical scrapie disease progression following exposure to the transmissible agent (for review see [12,13]). Genetic testing for the five most common haplotype alleles (i.e., ARR, ARQ, AHQ, ARH, and VRQ) is a key feature of scrapie eradication programs [14]. Management decisions depend on which of the 15 possible combinations of these paired *PRNP *haplotypes (i.e., diplotypes) are present in an animal [15]. *PRNP *diplotype scoring is further complicated by the presence of ARK and TRQ haplotypes, where K is lysine and T is threonine. Although infrequently observed overall, these haplotypes are important in some flocks [16]. When the known variation is accounted for, codons 136 and 171 each have multiple adjacent polymorphic sites and may encode up to four amino acids. This type of genetic structure has been recognized as a significant challenge for ovine *PRNP *DNA testing and assay design [17-19].

Codon variants at positions 136, 154, and 171 are not the only ones associated with scrapie resistance. An M112T variant on the ARQ haplotype has been associated with scrapie resistance in orally-inoculated Suffolk sheep in the U.S. [20]. Specifically, sheep with one or two copies of T_112_ARQ are resistant to development of classical scrapie when compared to those homozygous for M_112_ARQ. A M112I variant on the ARQ haplotype has also been reported, but it was not evaluated for association with disease [21]. M137T and N176K variants on the ARQ haplotype have been associated with scrapie resistance in intercranially-inoculated, orally-inoculated, and naturally-infected Italian Sarda breed sheep [22,23]. The existence of genetically resistant ARQ sheep raises the possibility of eradicating classical scrapie through genetic selection without using ARR rams. For example, selection of sheep with T_112_ARQ, AT_137_RQ, or ARQK_176 _alleles may be useful in purebred populations where ARR rams are rare or unavailable.

Other *PRNP *codon variants associated with disease resistance include those for atypical scrapie and experimental BSE challenge in sheep. Atypical scrapie differs from classical scrapie in the agent's properties, genetics, and epidemiology [24]. *PRNP *mutations associated with susceptibility to atypical scrapie include a L141F variant and a rare octapeptide repeat insertion [25-28]. Also, experiments with intravenous BSE challenge in sheep indicated that a P168L variant increased survival time [29]. Thus, eight *PRNP *codons and an octapeptide repeat have been associated with various forms of ovine prion disease, i.e. classical scrapie (codons 112, 136, 137, 154, 171, and 176), atypical scrapie (codon 141 and an octapeptide repeat insertion) and experimental BSE (codon 168). These variants are encoded by 12 single nucleotide polymorphisms (SNPs) and a 24 bp indel, and are surrounded by 32 other polymorphic sites. The prevalence of these polymorphisms in targeted flocks may influence the accuracy genetic testing, and ultimately, the management of scrapie eradication.

Our goal was four-fold: 1) to develop an adaptive DNA sequencing strategy for unambiguously determining the full length *PRNP *coding sequence for any sheep; 2) to produce a set of reference sheep DNAs for standardizing prion genetic testing; 3) to develop internally-controlled mass spectrometry (MS) assays that accurately score codons 112, 136, 141, 154, and 171; and 4) to establish a set of reference DNAs for discovering SNPs, estimating allele frequencies, and analyzing inheritance patterns.

## Results

### A DNA sequencing strategy for ovine PRNP

**Figure 1:**
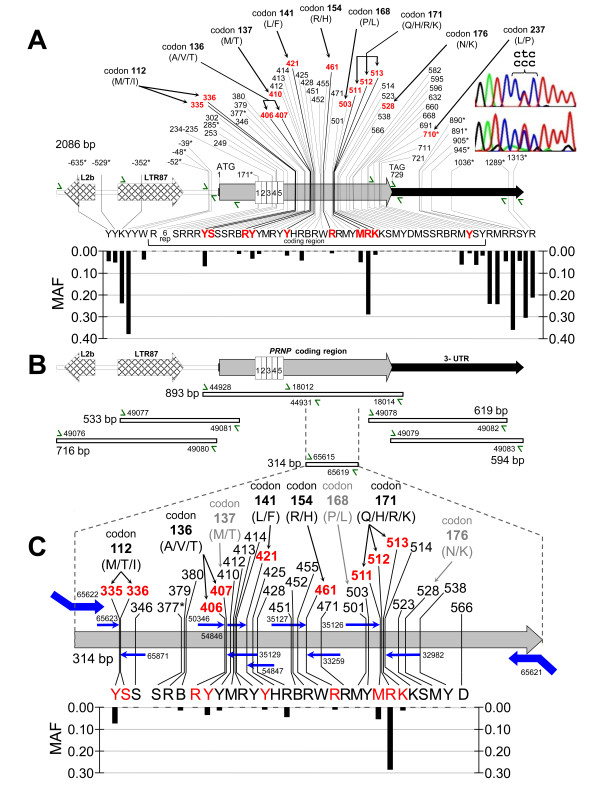
**Physical maps of the ovine *PRNP *coding sequence, polymorphisms, and assay elements**. Panel A features include: thick shaded arrow, coding sequence; black arrow, 3' untranslated region of exon 3; hatched arrows, ovine repetitive elements; white numbered vertical rectangles, octapeptide repeats; vertical lines, positions of SNPs; green single headed arrows, PCR amplification and/or sequencing primers (GenBank AY326330). SNP position numbers are distance to the first base of the *PRNP *start codon. Letters below SNPs are IUB ambiguity codes (R = a/g, Y = c/t, M = a/c, K = g/t, S = c/g, W = a/t, B = c/g/t, H = a/c/t, D = a/g/t) [56]. Red numbers and letters indicate sites affected by nonsynonymous substitutions at codons 112, 136, 154, 171, and 237. *PRNP *octapeptide repeats at positions 160 to 285 have either five or six repeats (5rep or 6rep). The asterisks denote SNPs not previously reported. MAF histograms correspond to genotypes from approximately 950 sheep available at http://cgemm.louisville.edu/USDA/index.html. Panel B: Map of ovine *PRNP *and regions targeted for PCR-amplification. PCR amplicons are depicted as open rectangles. The numbers by green arrows are USMARC primers (see Additional File 2). Panel C: Map of 314 bp PCR-amplified fragment for MALDI-TOF MS testing and expanded histogram of MAF. Features include: large bent blue arrows, PCR-amplification primers with mass tags added; horizontal blue arrows, hME extension primers for MALDI-TOF MS testing.

The objective was to develop an adaptive strategy whereby both the maternal and paternal alleles were evenly amplified and accurately scored for a given animal. The initial design accounted for the existence of 43 polymorphisms and depended on amplifying a "primary" *PRNP *amplicon and four additional overlapping amplicons (two each spanning either end of the primary amplicon; Figure 1A and 1B). Thus, unknown polymorphisms that could interfere with the amplification of the primary amplicon can be revealed in the sequences of overlapping amplicons and subsequently accounted for in additional rounds of PCR design. The primary DNA fragment was 893 bp in length and included the entire 771 bp coding region. There were no previously known polymorphisms in the primer binding sites of this amplicon that could otherwise interfere with faithful allele amplification.

**Figure 2:**
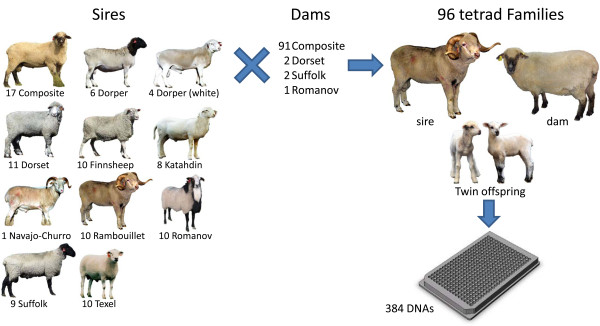
**USMARC Sheep Diversity Family Panel version 2.45**. The panel is composed of 96 unrelated sires, 96 ewes, and 192 twin offspring.

Sequence analysis of 192 reference sheep of diverse types (Figure 2) revealed a number of previously unreported SNPs, however none interfered with primer binding sites of the 893 bp amplicon. In the 953 sheep sequenced, 16 previously unreported SNPs were identified and 674 animals had at least one heterozygous site in the 893 bp PCR fragment. This result indicated that both alleles were amplified in those sheep. The remaining 279 sheep sequences contained no heterozygous sites. However, their homozygous diplotypes were tentatively inferred to be correct based on the lack of evidence for allelic dropout caused by a common SNP, and that 88% of these sheep had the two most common prion haplotypes: ARQ and ARR (haplotype frequencies of 0.598 and 0.288, respectively). However, if there are reasons to suspect allelic drop-out in sheep with homozygous diplotypes, the four overlapping amplicons may be sequenced in those sheep to verify the primer binding sites of their primary 893 bp amplicons.

Results from the group of 953 sheep presented here were combined with those available in the scientific literature [12,13] and GenBank [30] to produce a static composite consensus map for a 2086 bp region of *PRNP *that included 59 polymorphisms, 46 of which are in the coding sequence (Figure 1A). A dynamic map with breed frequencies, animal diplotypes, viewable trace files, and references was also produced and is available at: http://cgemm.louisville.edu/USDA/index.html.

### A novel L237P variant

**Figure 3:**
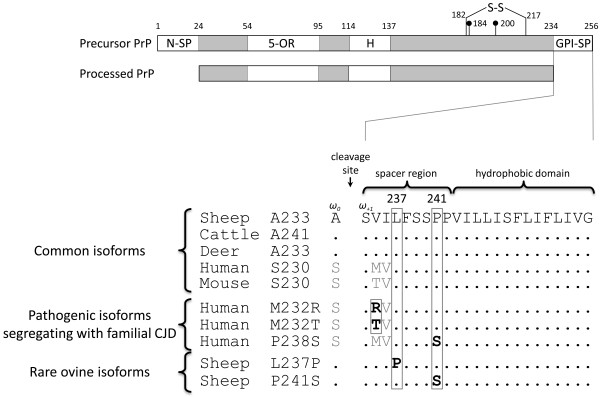
**Comparison of sequence variants in the PrP GPI-SP region**. Precursor PrP structural features include: an N-terminus signal peptide (N-SP), a five octapeptide repeat region (5-OR), a hydrophobic region (H), a disulfide bridge (S-S), N-linked glycosylation sites (dots), and a GPI signal peptide (GPI-SP). The residue numbers above the consensus sequence are those for ovine PrP. The peptide cleavage and GPI attachment site is indicated by omega-site zero (ω_0_). After synthesis and translocation to the endoplasmic reticulum, a GPI moiety is typically attached to the ω_0 _site of wild-type precursor PrP by a transamidation reaction and the last 23 residues are cleaved. The residues associated with familial CJD are shown in bold (M232R [34-37], M232T [33], P238S [38]). For comparison, nonsynonymous substitutions encoded by ovine *PRNP *are also shown in bold (L237P, this work; P241S [21,57]).

Of the 16 previously unreported *PRNP *SNPs, three were located in the coding region and one was predicted to alter the PrP amino acid sequence. This novel leucine (L)237proline (P) variant was discovered in a single composite ram while confirming its rare homozygous F_141 _diplotype. The most common alleles at position 237 encoded leucine (CTC, 0.95; CTG, 0.05). However the homozygous F_141 _ram was heterozygous at position 237 for a CCC allele encoding proline (Figure 1A). This polymorphism occurred in the highly conserved glycosylphosphatidylinositol (GPI) signal peptide (SP) region on C-terminus of the precursor PrP (Figure 3). The functional significance of a L237P variant is unknown.

### Frequencies of PRNP coding region polymorphisms

Whereas the consensus map depicts polymorphic loci from all reported testing, the allele frequency histogram (Figure 1A) depicts the amount of genetic diversity in the present group of 953 sheep. Using scores from Sanger sequencing trace files, the minor allele frequency (MAF) was calculated for all 59 polymorphisms in a 2086 bp region encompassing the *PRNP *coding region. Two features were noted. First, the MAFs of SNPs immediately adjacent to the coding region were generally higher than those in the coding region. Second, only one coding SNP (Q171R, nt 512) had a MAF greater than 0.100. The latter is consistent with the observation that many of these sheep were part of a scrapie eradication program in which R_171 _was selected for. In spite of the low overall MAFs estimated for most of the 46 coding region SNPs, the minor allele for any of these sites may cause significant scoring errors depending on the genetic history of the flock and the design of the DNA test.

### Frequencies of PRNP codon haplotypes

Haplotype and diplotype frequencies were tabulated for codon variants implicated in scrapie susceptibility and disease progression, i.e. at positions 112, 136, 141, 154, and 171 (Table 1). Codon 237 was also included in the analysis. Because the T_136 _allele was not observed in any animal, it was not included among the haplotype possibilities for these sheep. Nine haplotype phases were unambiguously established for the 883 animals because they were either homozygous, had only one heterozygous site, or were part of the 96 tetrad families depicted in Figure 2. The remaining 70 sheep had two heterozygous positions, e.g. ARR/AHQ. Haplotype phases were inferred for these 70 animals with the assumption that recombinant haplotypes were not present in these sheep. All 21 possible diplotype combinations of the six most common haplotype alleles at positions 136, 154, and 171 (i.e., ARQ, ARR, AHQ, ARH, VRQ, and ARK) were present in at least one animal in the group of 953 sheep. With the exception of ARQ, all haplotypes contained M_112 _and L_141 _alleles. Of the ARQ haplotypes, those with T_112 _were only observed in Suffolk, Rambouillet, and Composite rams; whereas those with F_141 _were only found in purebred Dorset rams or composite animals. When positions 112, 141, and 237 were included in the analysis, 36 of the 45 possible diplotypes were present in the group of 953 sheep. Individuals from this group of sheep were used to assemble a set of tissues and DNA representing standard *PRNP *diplotypes for DNA testing.

**Table 1:** Haplotype and diplotype frequencies for *PRNP *codons 112,136, 141, 154, 171, 237 in diverse samples of U.S. sheep

			Allele frequency														
			
Codon haplotype or diplotype				Panel version		Breeds on panels											
				
136 154 171	112 136 141 154 171 237	Genbank accession^a^	All USMARC sheep sequenced^b^	V1.1 V2.0 V2.4	V2.4	Composite	Dorset	Rambouillet	Texel	Suffolk	Finnsheep	Romanov	Katahdin	All Dorper	Dorper	White Dorper	Navajo-Churro
			(n = 953)	(n = 168)	(n = 96)	(n = 29)	(n = 18)	(n = 17)	(n = 18)	(n = 16)	(n = 19)	(n = 19)	(n = 15)	(n = 16)	(n = 8)	(n = 8)	(n = 1
ARQ	M**A**L**RQ**L	AY907689	0.517	0.534	0.522	0.385	0.270	0.457	0.194	0.729	0.868	0.921	0.533	0.719	0.750	0.688	0.500
ARQ	T**A**L**RQ**L	FJ404778	0.072	0.024	0.035	0.029	-^c^	0.014	-	0.021	-	-	-	-	-	-	-
ARQ	M**A**F**RQ**L	FJ404776	0.009	0.002	-	-	0.008	-	-	-	-	-	-	-	-	-	-
ARQ	M**A**F**RQ**P	FJ404776	0.001^d^	-	-	-	-	-	-	-	-	-	-	-	-	-	-

ARR	M**A**L**RR**L	AY907691	0.288	0.357	0.359	0.534	0.694	0.441	0.444	0.250	0.105	0.053	0.467	0.156	-	0.313	-
ARH	M**A**L**RH**L	AY907683	0.017	0.027	0.042	-	-	-	0.250	-	-	-	-	-	-	-	-
AHQ	M**A**L**HQ**L	AY909542	0.009	0.024	0.016	-	0.028	0.088	0.111	-	-	-	-	-	-	-	-
VRQ	M**V**L**RQ**L	AY907685	0.035	0.027	0.021	0.052	-	-	-	-	0.026	0.026	-	0.125	0.250	-	-
ARK	M**A**L**RK**L	EF189728	0.054	0.006	0.005	-	-	-	-	-	-	-	-	-	-	-	0.500
TRQ^d^	M**T**L**RQ**L^e^	AJ567987	-	-	-	-	-	-	-	-	-	-	-	-	-	-	-

ARQ, ARQ	MALRQL, MALRQL	AY907689	0.300	0.333	0.333	0.172	0.056	0.235	-	0.313	0.737	0.842	0.200	0.500	0.500	0.500	-
ARQ, ARQ	MALRQL, TALRQL	FJ404777	0.069	0.030	0.021	0.069	-	-	-	0.188	-	-	-	-	-	-	-
ARQ, ARQ	TALRQL, TALRQL	FJ404778	0.014	0.006	0.010	-	-	-	-	0.063	-	-	-	-	-	-	-
ARQ, ARQ	MALRQL, MAFRQL	EF189724	0.004	0.006	-	-	0.056	-	-	-	-	-	-	-	-	-	-
ARQ, ARQ	MAFRQL, MAFRQL	na^f^	0.001	-	-	-	-	-	-	-	-	-	-	-	-	-	-
ARQ, ARQ	MALRQL, MAFRQP	na	0.001^d^	-	-	-	-	-	-	-	-	-	-	-	-	-	-
ARQ, ARQ	MAFRQL, MAFRQP	FJ404776	0.008^d^	-	-	-	-	-	-	-	-	-	-	-	-	-	-
ARQ, ARQ	MAFRQP, MAFRQP	na	0.001^d^	-	-	-	-	-	-	-	-	-	-	-	-	-	-
ARQ, ARQ	MAFRQL, TALRQL	na	-	-	-	-	-	-	-	-	-	-	-	-	-	-	-
ARQ, ARQ	MAFRQP, TALRQL	na	-	-	-	-	-	-	-	-	-	-	-	-	-	-	-

ARQ, ARR	MALRQL, MALRRL	AY907684	0.254	0.262	0.271	0.276	0.333	0.412	0.111	0.125	0.211	0.105	0.667	0.188	-	0.375	-
ARQ, ARR	TALRQL, MALRRL	FJ404777	0.046	0.030	0.021	-	-	0.059	-	0.250	-	-	-	-	-	-	-
ARQ, ARR	MAFRQL, MALRRL	na	0.011	-	-	-	-	-	-	-	-	-	-	-	-	-	-
ARQ, ARR	MAFRQP, MALRRL	na	0.003^d^	-	-	-	-	-	-	-	-	-	-	-	-	-	-

ARQ, ARH	MALRQL, MALRHL	AY907693	0.015	0.024	0.042	-	-	-	0.222	-	-	-	-	-	-	-	-
ARQ, ARH	TALRQL, MALRHL	na	0.001	-	-	-	-	-	-	-	-	-	-	-	-	-	-
ARQ, ARH	MAFRQL, MALRHL	na	0.001	-	-	-	-	-	-	-	-	-	-	-	-	-	-
ARQ, ARH	MAFRQP, MALRHL	na	-	-	-	-	-	-	-	-	-	-	-	-	-	-	-

ARQ, AHQ	MALRQL, MALHQL	AY907687	0.002	0.006	0.010	-	-	-	0.056	-	-	-	-	-	-	-	-
ARQ, AHQ	TALRQL, MALHQL	na	-	-	-	-	-	-	-	-	-	-	-	-	-	-	-
ARQ, AHQ	MAFRQL, MALHQL	na	-	-	-	-	-	-	-	-	-	-	-	-	-	-	-
ARQ, AHQ	MAFRQP, MALHQL	na	-	-	-	-	-	-	-	-	-	-	-	-	-	-	-

ARQ, VRQ	MALRQL, MVLRQL	AY907690	0.029	0.042	0.031	0.034	-	-	-	-	0.053	0.053	-	0.250	0.500	-	-
ARQ, VRQ	TALRQL, MVLRQL	FJ404777	-	0.006	-	0.034	-	-	-	-	-	-	-	-	-	-	-
ARQ, VRQ	MAFRQL, MVLRQL	na	0.001	-	-	-	-	-	-	-	-	-	-	-	-	-	-
ARQ, VRQ	MAFRQP, MVLRQL	na	-	-	-	-	-	-	-	-	-	-	-	-	-	-	-

ARQ, ARK	MALRQL, MALRKL	EF189727	0.037	0.006	0.010	-	-	-	-	-	-	-	-	-	-	-	1.000
ARQ, ARK	TALRQL, MALRKL	na	-	-	-	-	-	-	-	-	-	-	-	-	-	-	-
ARQ, ARK	MAFRQL, MALRKL	na	-	-	-	-	-	-	-	-	-	-	-	-	-	-	-
ARQ, ARK	MAFRQP, MALRKL	na	-	-	-	-	-	-	-	-	-	-	-	-	-	-	-

ARR, ARR	MALRRL, MALRRL	AY907691	0.103	0.185	0.188	0.379	0.500	0.176	0.222	0.063	-	-	0.133	0.063	-	0.125	-
ARR, ARH	MALRRL, MALRHL	AY907681	0.005	0.018	0.021	-	-	-	0.167	-	-	-	-	-	-	-	-
ARR, AHQ	MALRRL, MALHQL	AY907682	0.008	0.030	0.021	-	0.056	0.059	0.167	-	-	-	-	-	-	-	-
ARR, VRQ	MALRRL, MVLRQL	AY907688	0.020	0.006	0.010	0.034	0.000	-	-	-	-	-	-	-	-	-	-
ARR, ARK	MALRRL, MALRKL	EF189729	0.026	-	-	-	-	-	-	-	-	-	-	-	-	-	-

ARH, ARH	MALRHL, MALRHL	AY907683	0.001	0.006	0.010	-	-	-	0.056	-	-	-	-	-	-	-	-
ARH, AHQ	MALRHL, MALHQL	AY907692	0.001	-	-	-	-	-	-	-	-	-	-	-	-	-	-
ARH, VRQ	MALRHL, MVLRQL	AY907694	0.001	-	-	-	-	-	-	-	-	-	-	-	-	-	-
ARH, ARK	MALRHL, MALRKL	EF189726	0.007	-	-	-	-	-	-	-	-	-	-	-	-	-	-

AHQ, AHQ	MALHQL, MALHQL	AY909542	0.001	0.006	-	-	-	0.059	-	-	-	-	-	-	-	-	-
AHQ, VRQ	MALHQL, MVLRQL	AY907686	0.001	-	-	-	-	-	-	-	-	-	-	-	-	-	-
AHQ, ARK	MALHQL, MALRKL	EF189725	0.004	-	-	-	-	-	-	-	-	-	-	-	-	-	-

VRQ, VRQ	MVLRQL, MVLRQL	AY907685	0.004	-	-	-	-	-	-	-	-	-	-	-	-	-	-
VRQ, ARK	MVLRQL, MALRKL	EF189722	0.008	-	-	-	-	-	-	-	-	-	-	-	-	-	-

ARK, ARK	MALRKL, MALRKL	EF189728	0.012	-	-	-	-	-	-	-	-	-	-	-	-	-	-

^a^GenBank accession numbers for sequences from control animals on MSCP27 with the relavant diplotype (where available).

^b^Sheep born 1995 to 2007. Allele or genotype frequencies that add up to 0.999 or 1.001 are the result of rounding errors.

^c^No minor alleles detected

^d^Sheep with 237P allele are descendents of a single composite ram

^e^Not present in U.S. populations surveyed. GenBank accession number correspends to Billinis et al. 2004. J. Gen. Virol. 85, 547-554

^f^Not available

### An ovine reference DNA panel for PRNP genetic testing

First, a set of tissues was assembled from 21 healthy sheep representing all diplotype combinations of the six most common haplotype alleles at positions 136, 154, and 171 (i.e., ARQ, ARR, AHQ, ARH, VRQ, and ARK; Table 2). Second, tissues of three additional sheep were included to represent the diplotype combinations of codon 112 (i.e., MM, MT, and TT) that occur on the ARQ haplotype. Third, tissues from sheep with four of six possible diplotype combinations of the three haplotype alleles known for codons 141 and 237 (i.e., haplotype alleles LL, FL, and FP) that occur on the ARQ haplotype. The variants of the ARQ haplotype were included because alleles at positions 112 and 141 have been implicated in scrapie resistance and are available in our populations. The two remaining tissue sets needed to complete this collection are expected to be produced in the spring of 2010 and available in the fall (i.e., MALRQL, MAFRQP and MAFRQL, MAFRQL; Table 2). Approximately 2 to 3 kg of DNA-rich tissues were collected from each animal sampled, thus providing a significant supply for wide-spread use. The complete *PRNP *coding sequence has been determined for each of the 28 animals and deposited in GenBank (Table 2). In addition, a set of 20 highly informative autosomal ovine SNPs were scored to provide a genetic "bar code" for tracking these samples within and between laboratories and resolve sample mix-up issues where they occur (Table 2).

**Table 2:** Composition and diplotypes of the USMARC Sheep *PRNP *Control Panel version 28

				*Prnp *codon genotypes^a^		Genotypes for sample identification^b^																			
					
GenBank **Acc. No**.	MARC **animal no**.	Breed	Sex	codons 136 154 171	codons 112 136 141 154 171 237	1^c^	2	3	4	5	6	7	8	9	10	11	12	13	14	15	16	17	18	19	20
AY907691	200423666	MARCIII Composite	ram	ARR, ARR	MALRRL, MALRRL	R	C	Y	T	R	W	G	T	G	R	G	K	W	G	R	G	Y	Y	R	C
DQ345759	200323140	MARCIII Composite	eve	ARR, ARQ	MALRRL, MALRQL	G	Y	Y	Y	A	T	T	Y	R	G	G	T	W	S	G	A	Y	C	R	Y
AY907682	199735002	Rambouillet	ram	ARR, AHQ	MALRRL, MALHQL	A	C	C	C	R	W	G	T	G	R	G	G	W	G	R	R	Y	C	G	T
AY907681	200450394	Texel cross	ram	ARR, ARH	MALRRL, MALRHL	R	T	Y	C	R	W	G	C	R	A	T	O	T	S	G	R	Y	Y	R	C
AY907688	200423500	MARCIII Composite	ram	ARR, VRQ	MALRRL, MVLRQL	A	Y	T	Y	A	T	G	C	R	R	K	G	W	S	A	R	Y	Y	G	T
EF189729	200665217	Navajo Churro cross	ewe	ARR, ARK	MALRRL, MALRKL	G	Y	Y	T	G	W	K	C	R	A	G	K	T	S	G	R	Y	C	A	Y
AY907689	200423510	MARCIII Composite	ram	ARQ, ARQ	MALRQL, MALRQL	R	C	C	Y	A	A	G	T	R	R	K	K	W	S	R	R	Y	T	A	Y
AY907687	200206039	Texel	ram	ARQ, AHQ	MALRQL, MALHQL	G	Y	Y	Y	R	A	G	Y	A	A	K	K	A	S	G	A	T	Y	A	C
AY907693	200450134	Texel cross	ram	ARQ, ARH	MALRQL, MALRHL	R	Y	Y	T	G	A	K	T	R	A	K	G	T	G	R	R	T	Y	G	C
AY907690	200423529	MARCIII Composite	ram	ARQ, VRQ	MALRQL, MVLRQL	R	T	Y	Y	G	T	K	C	R	R	T	G	A	C	A	R	T	Y	R	Y
EF189727	200665234	Navajo Churro cross	ram	ARQ, ARK	MALRQL, MALRKL	R	Y	Y	Y	G	W	G	Y	R	A	K	T	T	G	G	G	O	C	R	Y
AY909542	199935904	Rambouillet	ram	AHQ, AHQ	MALHQL, MALHQL	A	C	T	C	G	T	K	Y	G	R	K	K	A	C	G	R	Y	Y	A	Y
AY907692	200450114	Texel cross	ewe	AHQ, ARH	MALHQL, MALRHL	R	T	Y	C	R	W	G	Y	R	A	K	G	W	S	G	A	T	T	R	Y
AY907686	200177091	Rambouillet-Romanov	ewe	AHQ, VRQ	MALHQL, MVLRQL	R	Y	T	C	R	T	G	Y	R	G	T	G	W	S	G	R	Y	T	A	Y
EF189725	200665201	Navajo Churro cross	ram	AHQ, ARK	MALHQL, MALRKL	A	C	Y	Y	R	W	G	T	R	A	G	K	A	G	G	A	Y	C	R	Y
AY907683	200006024	Texel	ram	ARH, ARH	MALRHL, MALRHL	A	C	C	C	R	W	K	Y	G	A	T	K	A	G	G	A	O	C	R	C
AY907694	200450173	Texel cross	ewe	ARH, VRQ	MALRHL, MVLRQL	R	T	Y	Y	G	W	G	Y	G	A	T	G	W	S	G	R	Y	Y	R	C
EF189726	200665213	Navajo Churro cross	ram	ARH, ARK	MALRHL, MALRKL	A	Y	Y	T	G	T	G	C	A	A	K	T	A	G	R	G	O	C	A	Y
AY907685	200123331	MARCIII Composite	ewe	VRQ, VRQ	MVLRQL, MVLRQL	R	C	Y	Y	R	W	G	C	G	G	K	G	W	S	R	G	O	Y	R	C
EF189722	200665222	Navajo Churro cross	ewe	VRQ, ARK	MVLRQL, MALRKL	R	C	T	T	G	W	K	Y	R	R	K	K	A	G	G	G	Y	Y	R	Y
EF189728	200665931	Barbados Black Belly	ram	ARK, ARK	MALRKL, MALRKL	R	Y	C	C	R	T	G	Y	A	G	T	K	T	S	A	A	Y	Y	R	T

FJ404779	200117482	MARCIII Composite	ewe	ARQ, ARR	MALRQL, MALRRL	R	Y	C	Y	A	A	K	C	A	R	G	T	T	S	G	A	Y	C	A	T
FJ404777	200210034	MARCIII Composite	ewe	ARQ, ARQ	MALRQL, TALRQL	R	C	T	C	A	A	K	C	A	A	G	T	A	C	G	R	T	C	A	Y
FJ404778	200310125	MARCIII Composite	ewe	ARQ, ARQ	TALRQL, TALRQL	G	T	Y	Y	R	A	K	Y	R	A	K	T	A	G	A	R	C	C	A	Y

EF189723	200603590	Navajo Churro cross	ram	ARQ, ARQ	MALRQL, TALRQL	G	T	C	C	A	W	K	T	R	R	T	G	T	S	R	R	Y	Y	G	C
EF189724	200277004	MARCIII Composite	ewe	ARQ, ARQ	MALRQL, MAFRQL	R	Y	C	C	R	W	K	Y	G	A	G	T	A	C	R	G	Y	C	G	C
-^d^	-	-	-	ARQ, ARQ	MALRQL, MAFRQP	-	-	-	-	-	-	-	-	-	-	-	-	-	-	-	-	-	-	-	-
-	-	-	-	ARQ, ARQ	MAFRQL, MAFRQL	-	-	-	-	-	-	-	-	-	-	-	-	-	-	-	-	-	-	-	-
FJ404776	200865003	MARCIII Composite	ewe	ARQ, ARQ	MAFRQL, MAFRQP	R	T	C	T	R	W	T	C	A	A	G	G	W	S	R	R	Y	Y	R	T
GQ380576	200941201	MARCIII Composite	ram	ARQ, ARQ	MAFRQP, MAFRQP	-	-	-	-	-	-	-	-	-	-	-	-	-	-	-	-	-	-	-	-

^a^The predicted amino acid residues haplotypes inferred from those known to be segregating in these USMARC sheep populations.

^b^Autosomal SNP markers were selected from those reported by Kijas et al. (PLoS ONE. 2009;4(3):e4668). The genotypes were obtained by Sanger sequencing. Homozygous genotypes are listed as a single letter corresponding to the nucleotide at those sites, whereas, IUB ambiguity codes denote heterozygous genotypes those site: Y = C,T; R = A,G; M = A,C; K = G,T; W = A,T; S = C,G. Genotypes listed as "O" indicate missing information. The SNP marker properties and details are available at this site: http://cgemm.louisville.edu/USDA/index.html. Marker 250506CS is ISGC EST 250506CS3900218700001.

^c^Marker names: 1, CZ920950; 2, DU183112; 3, DU191809; 4, DU202116; 5, DU202534; 6, DU223430; 7, DU232778; 8, DU247686; 9, DU275655; 10, DU301854; 11, DU320019; 12, DU351298; 13, DU360304; 14, DU366451; 15, DU383209; 16, DU442796; 17, DU487949; 18, DU494996; 19, DU499024; 20, 250506CS3900218700001.

^d^Information expected to be available in fall of 2010.

### An internally-controlled homogeneous Mass Extend (hME)-type MALDI-TOF MS assay for codon testing

**Figure 4:**
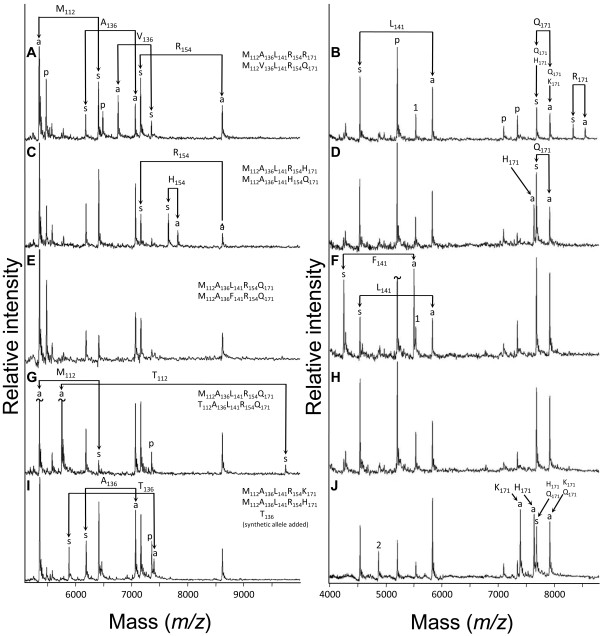
**Mass spectrograms of ovine *PRNP *codons at positions 112, 136, 141, 154, and 171**. A single PCR reaction was used to amplify a 336 bp genomic DNA region and the product split for use in two subsequent multiplex hME reactions. Spectral peaks represent singly-charged ions whose mass-to-charge ratio (*m*/*z*) was compared with calibrants for mass determination. Spectra feature labels: s and a, sense and antisense analytes produced from respective hME extension primers; p, unincorporated extension primer; ~, peak height clipped to conserve space. Two artifact peaks are produced as a consequence of multiplex design considerations. The first is a g nucleotide "pausing peak" in the codon 141 antisense assay (5530 Da, feature label "1"). The second artifact peak (feature label "2") is a g nucleotide misincorporation/insertion followed by a ddT termination in the codon 141 sense assay, i.e. 5'-[primer]-CGddT-3' (4866 Da). The correct termination product is 5'-[primer]-CddT-3' (4537 Da). This artifact peak at 4866 Da appears sporadically and independent of sample type or quality. Panels A and B: mass spectrograms illustrating the A136V and Q171R heterozygote. Panels C and D: mass spectrograms illustrating the R154H and Q171H heterozygote. Panels E and F: mass spectrograms illustrating the L141F heterozygote. Panels G and H: mass spectrograms illustrating the M112T heterozygote. Panels I and J: mass spectrograms illustrating the A136T and H171K heterozygote. The T136 was a synthetic allele that was added to the primer extension reaction cocktail to reference animal 200665213 (homozygous for A136).

A 314 bp *PRNP *fragment was amplified from genomic DNA for scrapie susceptibility testing. In addition to scoring the widely implicated codons 136, 154, and 171, our assay was designed to score codons 112 and 141 to facilitate investigation of these alleles in scrapie infected flocks. The 314 bp fragment had no known polymorphisms in its amplification primer binding sites (Figure 1C). Accurate codon diplotype scoring for multiple adjacent SNPs was achieved in two reactions where both the sense and antisense DNA strands were simultaneously scored in the same reaction. Codons 112, 136, and 154 were scored in one multiplex reaction, whereas codons 141 and 171 were scored in another (Figure 4). Although T_136 _was not present in the sheep tested, a synthetic DNA control for T_136 _produced good results when added to DNA amplified from homozygous A_136 _sheep. For codons 112, 136, 141, and 154, scoring from either DNA strand produced a complete diplotype. Thus, when both DNA strands were scored in the same reaction, concordance provided an internal diplotyping control. This was important because 15 other nearby SNPs were known to be present in eight of ten extension primer binding sites and may cause allele dropout in certain animals (Figure 1C). Scoring codon 171 required analysis of both sense and antisense strands to unambiguously infer the diplotype (Figure 4B, D, and 4J). In blind comparisons between diplotypes derived from Sanger sequence versus those from hME MALDI-TOF MS, 100% concordance was observed for the 28 sheep from the Scrapie Control Panel and the 192 parents from the Diversity Family Panel (data not shown). Together, these hME assays provide one example of well-characterized high-throughput MALDI-TOF MS assays for scoring *PRNP *codons 112, 136, 141, 154, and 171.

### Confirming relationships among 96 candidate families

A group of diverse rams were mated with ewes to produce families with twin lambs (i.e., tetrad families, Figure 2). Autosomal SNP diplotypes at 60 SNP loci were used to confirm relationships among sheep from 96 candidate tetrad families. These SNP loci included five from *PRNP *and 55 at other sites distributed across the genome (Additional File 1). Analysis of the 60 MALDI-TOF MS diplotypes for all 96 candidate families (i.e., 23,040 diplotypes) showed that Mendelian inheritance patterns were present in 94 of 96 families. Two families each had a single non-Mendelian inheritance pattern attributed to a distinct SNP. However, subsequent diplotypes scored from redundant Sanger sequencing revealed that the two MALDI-TOF MS diplotypes were incorrect. This error rate (two detected errors per 23,040 scored diplotypes) is well within the 99% accuracy expected for multi-plexed MALDI-TOF MS diplotype scoring and thus, the proposed family relationships in all 96 tetrad families appeared to be correct. The diverse group of sires for these families represents a minimal set of sheep and breeds for SNP discovery and allele frequency estimation. Their dams and offspring allowed haplotype phasing and verification of rare SNPs by allele segregation, features important for designing efficient and accurate DNA tests.

## Discussion

Commercial DNA testing technology has advanced rapidly during the past decade and access to services has increased significantly around the world. The ultimate promise of livestock DNA testing is to read an animal's DNA sequence at birth and make accurate predictions about its future performance. For traits where known DNA sequence variants have high predictive values, the challenge for DNA testing service organizations still remains: develop efficient, economical, and accurate commercial tests for livestock producers. Widely available and well-characterized sets of reference animals, tissues, DNA, and genetic information facilitate the development of new tests and routine monitoring of quality control. However, for many laboratories it is neither feasible nor desirable to assemble a proprietary collection of reference samples. For example, the most efficient way to obtain some *PRNP *diplotype combinations is by mating numerous rare sheep. The present report describes an adaptive *PRNP *DNA sequencing strategy for any sheep population, a set of 28 reference DNAs for standardized *PRNP *genetic testing, high-throughput assays for scoring five important *PRNP *codons, and a family-based set of 384 reference DNAs for discovering SNPs, estimating allele frequencies, and analyzing inheritance patterns. These are publicly available without restriction for ovine scrapie susceptibility testing, training, and research.

The ovine prion gene is likely the most sequenced gene in sheep, and novel SNPs with low MAF are routinely discovered. The present strategy of sequencing overlapping amplicons for a 2 kb region of ovine *PRNP *identified 16 previously unreported SNPs in 953 sheep (1 SNP per 130 bp of consensus sequence). This approach was similar to that used for a 25 kb region of bovine *PRNP *where 287 novel SNPs were discovered in 192 beef and dairy cattle (1 SNP per 88 bp of consensus sequence) [31,32]. The 16 previously unreported ovine SNPs included a L237P variant in the GPI-SP region of PrP on the F_141 _haplotype. This result is intriguing because human mutations in this region segregate with CJD [33-38] and the ovine F_141 _haplotype is strongly associated with atypical scrapie [26-28,39] (Figure 3). Surveys of atypical scrapie have shown that 103 of 241 cases (43%) contain one or two copies of F_141 _[24]. Although the status of codon 237 was not included in these reports of atypical scrapie, it would be useful to know if classifying the F_141 _haplotype into subtypes F_141_L_237 _or F_141_P_237 _affects the strength of association. In addition to *PRNP *genetic testing, genomic DNA now available from these and other haplotypes reported here may be useful for cloning specific *PRNP *haplotypes for *in vitro *or *in vivo *experiments aimed at testing the relative effects of particular PrP isoforms.

During the last ten years, more than a dozen reports of ovine *PRNP *genetic testing systems have been described, including some that employed MALDI-TOF MS technology [17-19,40-52]. The MALDI-TOF MS multiplex assays described in this report provide an enhanced multiplex design that includes alleles not previously tested, scores alleles from both DNA strands in the same reaction, and accounts for newly recognized nearby polymorphisms. This assay design may be useful for comparisons with other testing platforms or as a starting point from which to tailor genetic testing needs to specific populations. In addition to the ovine *PRNP *SNPs tested here, other polymorphisms have also been associated with prion disease susceptibility, e.g. codons 143, 168, 176, and an octapeptide repeat insertion [22,23,28,29]. Thus, MALDI-TOF MS assays presented here are an example of one design where others are possible.

Lastly, this report describes a well-characterized set of 96 tetrad families that can be used for routine SNP discovery, validation, and haplotype phase determination. Its use allows confirmation of potentially complex multi-locus haplotypes to be resolved by segregation analysis. Although we have employed it specifically for analyzing the *PRNP *gene, it is generally applicable to any gene or region of the ovine genome.

## Conclusion

The ability to identify *PRNP *polymorphisms in any sheep provides critical information for designing efficient population-based scrapie genetic testing systems. Combined with reference DNA panels, these resources facilitate training, certification, and the development of new tests and knowledge that may expedite the eradication of sheep scrapie.

## Methods

### Animals, health status, and tissue collection

All animal procedures were reviewed and approved by the USMARC Animal Care and Use Committee prior to their implementation. Because health status is important for providing tissues and purified DNAs to an international community, tissues were collected from healthy sheep, i.e., without signs or history of clinical disease. Since first stocking sheep in 1966, USMARC has not had a known case of scrapie. Until 2002, surveillance consisted of monitoring sheep for possible signs of scrapie and submitting brain samples to the USDA Animal and Plant Health Inspection Service (APHIS) National Veterinary Services Laboratory in Ames, IA for testing. All tests have been negative. Since April 2002, USMARC has voluntarily participated in the APHIS Scrapie Flock Certification Program, is in compliance with the National Scrapie Eradication Program, and is certified as scrapie-free. However, it is recognized that the USMARC flock of 4000 breeding ewes is currently located in a bluetongue medium incidence area and is known to harbor some levels of contagious ecthyma, foot rot, paratuberculosis (Johne's disease), ovine progressive pneumonia (OPP) and pseudotuberculosis caseous lymphadenitis.

When samples were collected for limited use, whole blood (8 ml) was drawn in commercially prepared EDTA tubes (Sarstedt Inc., Newton, NC, USA). For research applications where extended use was anticipated, whole blood (150 to 300 ml) was drawn in syringes containing 1% vol/vol sterile molecular biology grade 0.5 M EDTA pH 8.0 (USB Corporation, Cleveland, OH, USA). For the Scrapie Control Panel DNA collection, sheep were euthanized at USMARC and DNA-rich tissues were collected: whole blood (~300 ml), liver (~900 g), lung (~800 g), kidney (~125 g), and spleen (~125 g). All samples were stored at -20°C until DNA was extracted.

### DNA extraction and Sanger sequencing

DNA from freeze-thawed whole blood samples (200 μl) was extracted by use of a solid-phase system incorporating either spin-columns or 96-well microtitration plates according to the manufacturer's instructions (Gentra Systems, Inc., Minneapolis, MN, USA). DNA from 5 ml blood samples or solid tissues was extracted by standard procedures that use a mixture of phenol, chloroform, and isoamyl alcohol to remove proteins and other contaminants [53]. Purity and amount of DNA was estimated spectrophotometrically by the ratio of absorptions at 260 nm versus 280 nm (NanoDrop products, Wilmington, DE, USA) and compared to double stranded DNA measurements with PicoGreen dsDNA Reagent per manufacturer's instruction (Invitrogen Corporation, Carlsbad, CA, USA). Polymerase chain reaction (PCR) cocktails and DNA sequencing reactions were carried out as previously described [53]. The oligonucleotides for ovine *PRNP *amplification and DNA sequencing are provided in Additional File 2. Both strands of each amplicon were sequenced for each animal to increase the quality of their consensus sequence. The DNA sequences, allele frequencies, SNP diplotypes of animals, and their tracefiles are publicly available at: http://cgemm.louisville.edu/USDA/index.html.

### Assembly of an ovine reference DNA panel for PRNP genetic testing

The USMARC Sheep *PRNP *Control Panel version 28 consisted of 13 rams, three wethers, and 12 ewes representing three distinct sets of reference animals: 1) all 21 possible diplotype combinations from the six most common *PRNP *haplotype alleles (i.e., ARR, ARQ, AHQ, ARH, VRQ, and ARK) at codons 136, 154, and 171; 2) all three diplotype combinations of codon 112 (i.e., MM, MT, and TT); and 3) four of six possible diplotype combinations of the three haplotype alleles known for codons 141 and 237 (i.e., haplotype alleles LL, FL, and FP).

### Sheep Diversity Panels for SNP discovery and allele frequency estimation

Three sequential versions of USMARC Sheep Diversity Panels were used. The purpose of these panels was SNP discovery and allele frequency estimation. The first panel version (1.1, [54]) consisted of 90 rams from nine breeds (Dorper, White Dorper, Dorset, Finnsheep, Katahdin, Rambouillet, Romanov, Suffolk, and Texel) and a composite population (USMARCIII: 1/2 Columbia, 1/4 Hampshire, and 1/4 Suffolk [55]). These breeds were selected to represent genetic diversity for traits such as fertility, prolificacy, maternal ability, growth rate, carcass leanness, wool quality, mature weight, and longevity. The ten rams sampled from each breed were chosen to minimize genetic relationships among rams within breed. The second version (2.0) consisted of 96 rams from nine breeds and the composite population and was based on the same design as version 1.1. However, version 2.0 contained 78 rams not present on version 1.1. The third version (2.4) consisted of 95 rams from nine breeds and the composite population, plus one Navajo-Churro ram with a rare prion haplotype allele (ARK). The version 2.4 panel design is based on that of version 2.0, but contained five rams not present on version 2.0, and 78 rams that were not present on version 1.1. The 96 rams of version 2.4 sired twin offspring with known ewes, and are thus part of the 384-member USMARC Sheep Diversity Family Panel version 2.45.

### A family-based panel for validating SNPS and determining haplotype phase

The USMARC Sheep Diversity Family Panel version 2.45 consisted of the same 96 rams from the Sheep Diversity Panel version 2.4 (described above) mated to 91 USMARCIII ewes, two Dorset ewes, two Suffolk ewes, and a Romanov ewe to produce 192 non-identical twins in 96 tetrad families.

### Ovine PRNP matrix-assisted laser desorption/ionization time-of-flight (MALDI-TOF) MS assays

Efficient and accurate codon scoring is challenging when multiple adjacent SNPs are present in the codon. For example, the International Union of Biochemistry (IUB) ambiguity codes for the nucleotide consensus sequence for ovine *PRNP *codon 171 are "MRK", which represents these known codons at position 171: CAG (Glu), CGG (Arg), CAT (His), and AAG (Lys). One solution to this problem is to employ primer extension chemistry whereby an oligonucleotide primer binds to an adjacent sequence on each strand and synthesis DNA polymerase is used to extend the primer across one, two, or three SNPs with specific mixtures of deoxy- and dideoxynucleotides (dNTPs and ddNTPs). The advantage over chemistries that employ only ddNTPs and are designed to extend exactly one nucleotide, is the mass of extended oligonucleotides generated from dNTPs and ddNTPs provides information about the haplotype status of the alleles. When both DNA strands from both alleles are interrogated in the same reaction, their respective results must be consistent if they are to be believed. This provides a convenient control that is internal to the biochemical reaction. The oligonucleotides for ovine *PRNP *amplification and MALDI-TOF MS testing are provided in Additional File 2.

## Competing interests

All authors, except TSK, declare that they have no competing interests. TSK is the President and principal owner of Intrepid Bioinformatics Solutions, Inc., a company that develops data brokerage systems in support of life sciences research.

## Authors' contributions

All authors participated in interpreting the results, manuscript editing, and have read the final manuscript. In addition, MPH conceived the experimental design, identified sources of rare germplasm, designed DNA sequencing and genetic testing strategies, and prepared the manuscript. KAL created mating designs to produce rare diplotypes, co-designed sheep diversity and family panels, and collected tissues. BAF co-designed and tested sheep diversity panels. TPLS supervised the DNA sequencing. TSK carried out the design, development, and transfer of the *PRNP *trace data and genetic information to publicly accessible web sites. MLC participated in experimental design and tissue collections, and WWL participated in the experimental design.

## Supplementary Material

Additional file 1 **Family structure and diplotypes of the USMARC Sheep Diversity Family Panel version 2.45**. The diplotypes were obtained by at least two independent methods including MALDI-TOF MS with Sequenom iPLEX chemistry, Illumina BeadArray chemistry, and Sanger sequencing. Click here for file

Additional file 2 **Oligonucleotides for ovine *PRNP *amplification, DNA sequencing, and MALDI-TOF MS testing**. List of genotyping reagents and related assay information Click here for file
